# Self-Calibratable Absolute Modular Rotary Encoder: Development and Experimental Research

**DOI:** 10.3390/mi15091130

**Published:** 2024-09-05

**Authors:** Donatas Gurauskis, Dragan Marinkovic, Dalius Mažeika, Artūras Kilikevičius

**Affiliations:** 1 Department of Information Systems, Vilnius Gediminas Technical University, LT-10105 Vilnius, Lithuania; donatas.gurauskis@vilniustech.lt (D.G.); dalius.mazeika@vilniustech.lt (D.M.); 2 Department of Structural Analysis, TU Berlin, 10623 Berlin, Germany; 3 Institute of Mechanical Science, Vilnius Gediminas Technical University, LT-10105 Vilnius, Lithuania; arturas.kilikevicius@vilniustech.lt

**Keywords:** optical encoder, self-calibratable encoder, angular accuracy

## Abstract

Advanced microfabrication technologies have revolutionized the field of reflective encoders by integrating all necessary optical components and electronics into a miniature single-chip solution. Contemporary semiconductor sensors could operate at wide tolerance ranges that make them ideal for integration into compact and lightweight modular encoder kit systems. However, in order to achieve the high accuracy of the operating encoder, precise mechanical installation is still needed. To overcome this issue and exploit the full potential of modern sensors, the self-calibratable absolute modular rotary encoder is developed. The equal division average (EDA) method by combining the angular position readings from multiple optical sensors is used to simplify the installation process and ensure the high accuracy of the system. The produced prototype encoder is experimentally tested vs. the reference encoder and the measurement deviations of using different numbers and arrangements of optical sensors are determined. The obtained results show encoder ability to handle the mounting errors and minimize the initial system deviation by more than 90%.

## 1. Introduction

In the rapidly evolving landscape of automation and precision engineering, the demand for accurate and reliable angular motion sensing technologies has never been so high. Leading this technological frontier is the optical encoder. This device is a critical component in various high-tech industrial applications including servosystems and robotics, medical devices, radar, and tracking systems [1,2,3,4,5,6], as well as in aerospace and military applications.

**Figure 1:**
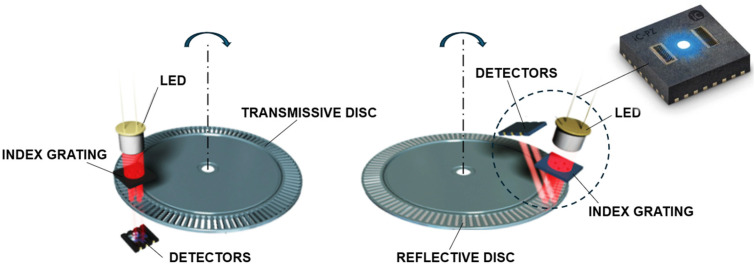
Working principle schemes of the transmissive and reflective optical encoders.

Two principal operation schemes are shown in Figure 1. The traditional transmissive optical encoder works by having a collimated light source and a stationary index grating placed on one side of a rotating code disc and a detector placed on the opposite side. The disc has a pattern of transparent and opaque segments that interrupts the light beam as it rotates. The detector senses the interruptions, converting them into electrical signals that represent the rotational position and the speed. In turn, the reflective optical encoder works by having a light source, index grating, and a detector on the same side of the code disc, which has a pattern of reflective and non-reflective segments. As this disc rotates, the emitted light reflects off the reflective segments back to the detectors, while the non-reflective segments scatter the light. The single-sided arrangement of these optical components simplifies the alignment process during the installation and makes the design compact. Moreover, microfabrication, which involves the miniaturization of devices through advanced manufacturing techniques, has revolutionized the field of reflective optical encoders by enabling ultra-precise single-chip solutions. The process that utilizes photolithography, etching, and deposition methods allows one to produce the fine-scale structures necessary to detect rotational position and velocity with exceptional accuracy. Modern customized optical sensors integrate not only LED, index grating, and the matrix of photodetectors, but also an analogue signal conditioner, interpolator, and other electronics. Such miniature semiconductor chips may have dimensions of 3 mm × 3 mm, work on air gaps from the code disc up to 3 mm, and handle tangential and radial misalignments of ±0.5 mm. This wide field of mechanical alignment makes them ideal for integration into a modular encoder kit system, whose bearingless and wear-free design has a huge benefit compared to conventional closed type encoders.

Nevertheless, precise mechanical installation is inevitable if the high accuracy of the operating encoder is needed. Any axial or radial runout of the application shaft as well as the eccentricity of the code disc must be considered in order to minimize the measurement error. On the other hand, various error compensation methods could be applied to overcome this issue. The typical error compensation practice involves the detection of angular position deviations and the corresponding correction of the encoder readings. The most common is the cross-calibration procedure, during which the encoder position readouts are compared to the readings of a reference [7,8,9,10,11,12,13]. In terms of open-type modular encoder systems, this method is not very convenient, because the encoders cannot be calibrated by the manufacturer. The procedure must be performed after the installation and requires a reference device that must be mechanically coupled with the encoder under the test.

In contrast, self-calibration methods do not require an external reference device, making them highly practical for in situ applications. The time measurement dynamic reversal (TDR) method is based on the encoder pulse width measurement in the time domain [14,15]. Since the angular deviations are estimated from the free response dynamics, the shaft of the application has to rotate at a certain speed and slow down freely. This method enables in-place calibration and gives error maps for further compensation. While effective, this method is dependent on specific operational conditions that may limit its applicability in all scenarios. The coaxial sensor relative rotation (CSRR) method allows the self-calibration of the encoder on the application shaft [16]. However, it requires the modification of the machine so that two sensors can rotate in locked and separate regimes at a constant speed, potentially complicating its implementation in existing systems.

Despite the attempts to develop the self-calibration techniques mentioned above, placing multiple optical sensors around the scale grating and calculating the compensated encoder position from the collective sensor readings remains the most reliable and effective method. The code disc eccentricity error elimination by using two sensors arranged at 180 degrees was proposed in [17,18]. Expanding this approach, the possibility of minimizing other mounting inaccuracies, such as code disc inclination, by using four equally spaced sensors was analysed in [19]. This configuration not only addressed eccentricity errors but also enhanced the overall system accuracy by effectively compensating for additional mounting imperfections. To achieve even higher accuracy and compensate for manufacturing errors of the code disc, several methods involving multiple optical sensors have been developed. In general, these methods could be categorized in two groups based on sensor distribution: uniform and non-uniform. Uniform distribution involves placing sensors at equal intervals around the encoder scale grating, which simplifies error compensation and is particularly effective in reducing harmonics and systematic errors. Among the uniform distribution methods, the equal division average (EDA) method is the most widely adopted and has been employed to develop super-high-resolution encoders using different types and numbers of reading heads [20,21,22,23,24]. EDA-method-based optical encoders are integrated into various high-precision systems, including high-accuracy calibration systems for angular encoders [25], the angular index table [26], the precision angle comparator [27], the angular velocity calibration system [28], and the portable real-time angle encoder [29].

On the other hand, non-uniform distribution strategically positions sensors at varying intervals around the rotary grating. The non-uniform distribution methods, such as the virtual equal division average (VEDA) [30,31] or the approach described by Tadashi Masuda et al. [32], offer the ability to detect and eliminate higher-order harmonic components by using less optical sensors. However, these methods often involve more complex calculation algorithms, which can be computationally intensive and challenging to implement in optical encoders requiring real-time operation. Furthermore, recent research [33] by V. P. Kiryanov et al. showed that uniform distribution methods, such as EDA, consistently achieve higher accuracy compared to non-uniform approaches. In addition, advancements in microfabrication techniques have significantly reduced the cost of optical sensors, making the integration of additional devices a cost-efficient solution. This economic feasibility enhances the appeal of adding multiple sensors to improve measurement accuracy, even when accounting for the increased complexity and processing requirements. Thus, the uniform distribution method not only provides higher accuracy but also remains practical and scalable for widespread industrial applications.

In this work, the self-calibratable absolute modular optical encoder based on the EDA method was developed. The experimental research by calibrating the produced prototype encoder versus the high-accuracy reference encoder was performed to practically evaluate the feasibility and efficiency of the EDA method in calibrating mounting errors. The obtained cross-calibration data of angular position deviations of different sensors were used to determine the actual accuracy of the proposed measurement system, which might be composed of different numbers and alignments of optical sensors. 

## 2. Overview

### 2.1. Working Principle of Optical Encoders

**Figure 2:**
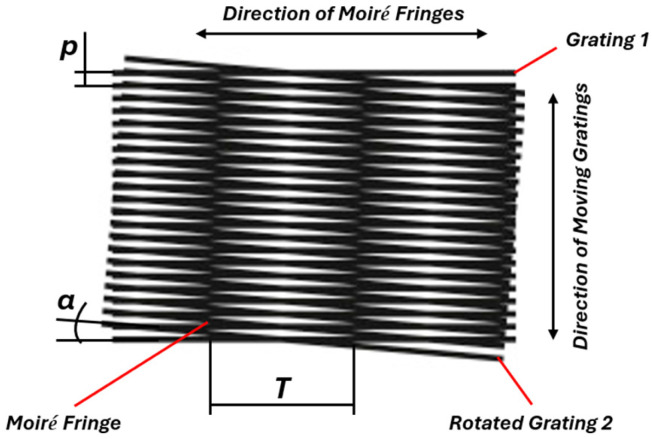
Superposition of two gratings and the formation of Moiré fringes.

The core principle behind an optical encoder is the use of light to detect changes in position by reading patterns on a disc. The motion of a moving pattern is converted into electrical signals, which can be read by a control system to determine position, speed, or direction. Various optical principles and effects are used to form high-quality signals that are the basis for the measurement of mechanical motion. One of the most widely used visual phenomenon is the Moiré effect. This occurs when two repetitive gratings are overlaid at an angle or with slight misalignment. This superposition leads to the formation of an interference pattern called Moiré fringes that remain a topic of significant interest across multiple fields, from fundamental research in physics to practical applications in engineering. Moiré fringes are particularly useful for analysing the structural properties of materials at the atomic scale in Transmission Electron Microscopy (TEM) [34,35,36], the strain measurement in materials and structures [37,38,39], the creation of a detailed 3D profile of a surface in computer-generated Moiré profilometry [40,41,42,43], or precise alignment of semiconductor layers in nanoimprint lithography (NIL) [44,45,46]. Moiré fringes in optical encoders appear when the collimated light passes through the stationary index grating and then encounters the second grating formed on the rotating disc. Observed fringes are directly influenced by the relative position and geometrical parameters of both gratings. The specific combination of these parameters results in a magnification effect, meaning that even a small rotation of the disc will cause a significantly larger displacement of the Moiré fringes [47,48,49,50]. The formation of a Moiré pattern from the superposition of two gratings with the same pitch *p* is illustrated in Figure 2. When the index grating is slightly rotated to form an angle α between gratings, the spacing *T* of the formed Moiré fringes could be expressed as follows [51]:

(1) $$T=\frac{p}{2sin\left(\frac{\alpha }{2}\right)},$$

The observed interference pattern occurs when it is detected by relatively placed photodetectors that forms the electrical signals. The intensity of light reaching the photodetectors varies according to the movement of the Moiré fringes, which corresponds to the rotation of the disc.

Despite the widespread use of the Moiré effect in optical encoders, other methods are also employed, such as the Lau effect, Talbot effect, generalized grating imaging, or interferometry. In a Talbot-effect-based optical encoder, a periodic grating, when illuminated by coherent light (usually a laser), produces self-image replicas at specific distances along the optical axis. These self-images repeat periodically and could be detected by photodetectors placed at these specific distances known as “Talbot” [52,53,54]. The Lau effect is similar to the Talbot effect but involves the superposition of multiple periodical gratings. When coherent light passes through two or more gratings, the resulting interference pattern can be observed and detected [55,56]. Encoders using generalized grating imaging involve sophisticated grating designs that can manipulate light to produce enhanced interference patterns, which are then analysed to extract positional information [57,58,59]. Interferometry involves the superposition of light waves, typically from a coherent source, to produce an interference pattern. The relative phase shifts of the interfering beams can be used to measure very small displacements with high accuracy [60,61,62].

### 2.2. Errors in Modular Optical Rotary Encoders

Open-type modular rotary encoders generally consist of a stationary reading head mounted separately from a code disc that is directly attached to the rotating shaft of the application. The open design means there is no integral bearings, which allows for a more compact and lightweight solution but also increases the encoder’s susceptibility to installation accuracy. In general, errors in modular rotary encoders can be classified into low-frequency and high-frequency errors, depending on their characteristics and the frequency at which they occur. The following subsections introduce both types of errors and the factors that contribute to them.

#### 2.2.1. Low-Frequency Errors

Low-frequency errors occur at frequencies that are typically associated with the rotation of the encoder and are often linked to mechanical issues in the system. Particularly important is the eccentricity and inclination errors of the code disc [63]. These mounting-related errors occur during the installation of the encoder’s rotor (disc-hub assembly) and are also influenced by the dimensional and form inaccuracies, as well as the radial runout of the application shaft.

**Figure 3:**
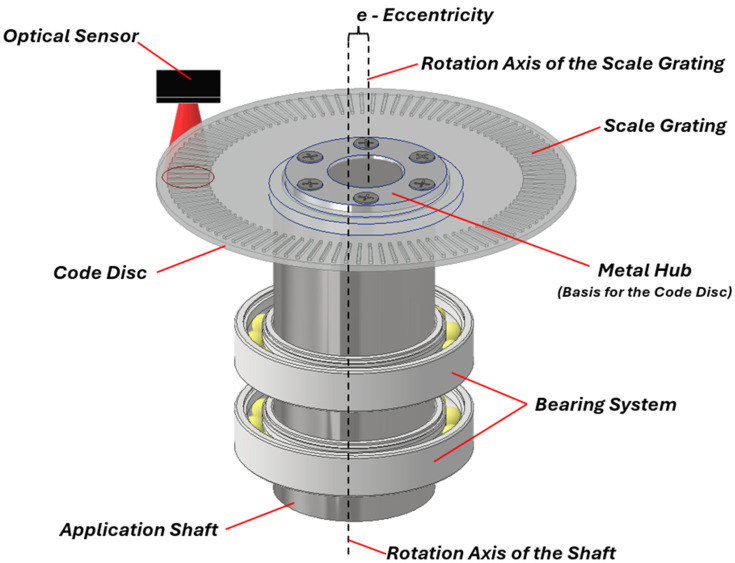
Presentation of scale grating installation with eccentricity error.

The eccentricity error is a periodic positioning error over one full revolution of the rotary axis. It appears when the glass disc of the encoder is mounted non-concentrically to the rotation axis of the measured shaft. The graphical presentation of the encoder rotor mounted with eccentricity is shown in Figure 3. The relationship between the scale grating eccentricity *e* (measured in µm), the mean diameter of the scale *D* (measured in mm), and the measurement error ∆φ (measured in arcseconds) can be expressed as follows:

(2) $$∆\varphi =\pm 412\frac{e}{D},$$

It is easy to evaluate that an eccentricity of 5 µm on a 65 mm mean diameter code disc grating results in an angular measurement error of approximately ±32 arcseconds. This demonstrates how even small physical displacement can lead to significant angular errors in open-type optical encoders.

Recent scientific research suggests several innovative approaches to deal with eccentricity errors in optical encoders. Xuan Li et al. proposed a prominent method that involves eccentricity self-detection using an advanced encoder equipped with a spider-web-pattern scale grating, which is scanned by a dual-head scanning unit [64]. This unit scans the grating disc in both angular and radial directions, allowing for real-time detection and the correction of eccentricity errors. Hua-Kun Jia et al. introduced a method based on a visual system and image processing technology to detect eccentricity [65]. However, implementing these methods requires significant changes to the encoder design, making them unsuitable and not cost-effective for many applications. Eliminating encoder eccentricity by using two optical sensors placed at an angle of 180 degrees to each other remains the most effective and reliable approach [17,18].

**Figure 4:**
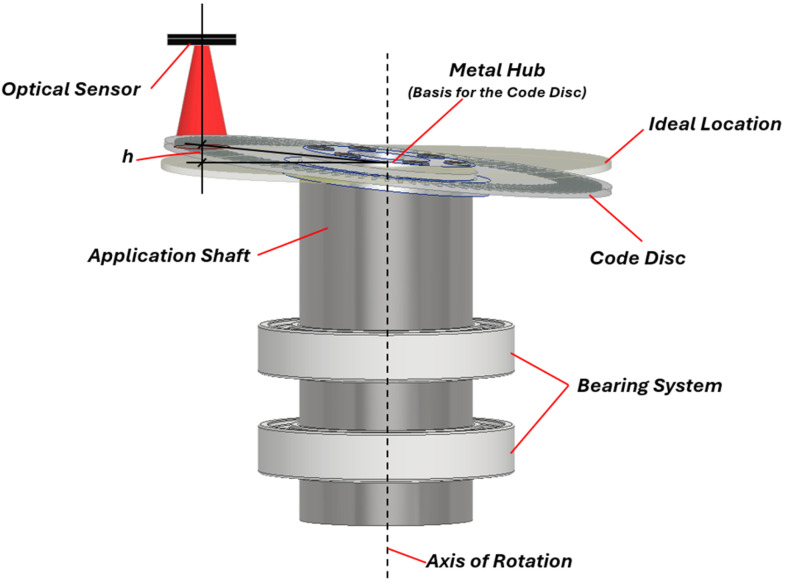
Presentation of scale grating installation with inclination error.

The code disc inclination error in optical rotary encoders occurs when the disc is not perfectly perpendicular to the rotational axis. This issue can arise due to the incorrect installation of the grating disc on the shaft, potentially caused by the improper seating of the encoder rotor or inconsistencies in the mounting surface. An example of incorrect disc-hub installation on the measured shaft is shown in Figure 4. As the shaft rotates, the disc may begin to swash, leading to a varying vertical deviation *h* from its nominal position. This swashing motion alters the total air gap between the disc and the optical sensor, which can cause fluctuations in encoder readings and result in measurement inaccuracies. A recent study by Hai Yu et al. analysed the grating disc inclination error in photoelectric encoders and its elimination by using multiple optical sensors [66]. The obtained results revealed that in addition to eccentricity errors, the tilt of the grating disc significantly affects measurement accuracy and cannot be ignored during the installation process. The inclined disc introduces an angle measurement deviation characterized by a “double cycle” fluctuation over 360 degrees. To effectively eliminate this error, at least three equally spaced optical sensors must be used.

Other low-frequency errors could be related to inaccuracies in the grating pitch and shape. Variations in the spacing of the grating lines on the encoder disc cause the electrical signal to deviate periodically as the disc rotates, typically occurring at harmonics of the rotational frequency, depending on the pitch variation. Additionally, a non-circular shape of the grating can lead to low-frequency errors. These shape errors could result from inaccurate manufacturing or from deformations of the glass disc introduced during assembly, such as those caused by hub-fixing screws.

#### 2.2.2. High-Frequency Errors

High-frequency errors occur at much higher frequencies and are often related to electronic or optical issues within the encoder system. Sub-division errors, signal noise, or mechanical vibrations typically occur at frequencies much higher than the primary rotational frequency. Sub-division errors in optical encoders refer to inaccuracies that arise during the analogue signal interpolation process, which is used to divide the signal into finer increments to achieve higher resolution. The signals provided by the photodetectors are processed by an electronic circuit to produce two sinusoidal electric signals. To calculate the finer position, these signals are typically analysed using an arctangent algorithm. For accurate results, the algorithm requires several specific conditions: the amplitudes of both signals must be constant and equal, there must be no offset, the phase shift between the signals must be precisely 90 degrees, and they must have perfectly sinusoidal waveforms [67]. Despite imperfections in the interpolation electronics, disturbances in the optical system are the primary source of non-ideal electrical signals. Any imperfections in the optical path, such as lens aberrations or alignment issues, can cause inaccuracies in the light intensity reaching the photodetectors. Additionally, any unwanted relative motion between the optical components, such as tilt or rotation of the index grating caused by mechanical deformations or vibrations, can result in the distorted superposition of the gratings and lead to unpredictable changes in Moiré fringes. These factors contribute to electrical signal distortions and result in metrological errors within the encoder system. Electrical noise arising from power supplies, electromagnetic interference (EMI), or signal cross-talk can introduce high-frequency errors into the encoder output signals. The frequency of this noise depends on the source, but it is generally high relative to the encoder operating frequency. Such a noise can distort the signals, leading to random fluctuations in the position measurement.

## 3. Materials and Methods

### 3.1. Development of the Sefl-Calibratable Optical Modular Encoder

In this subsection, the development of the prototype optical encoder for experimental research is described in more detail. The main mechanical and electrical parameters are discussed below.

#### 3.1.1. Mechanical Design of the Encoder

**Figure 5:**
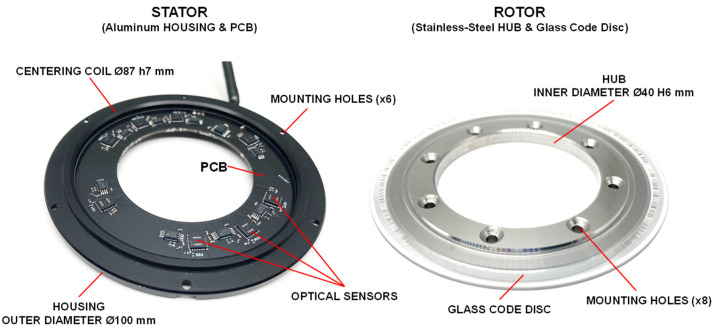
View of developed modular optical encoder.

The open-type encoder consisted of two main parts: a stator and rotor, as can be seen in Figure 5. The stator was made of aluminium housing with an attached printed circuit board (PCB). The PCB contained eight absolute optical sensors iC-PZ (made by “iC-Haus”, Bodenheim, Germany) and other electronics. The outer diameter of the stator was Ø100 mm, and it had a centring coil of Ø87 h7 mm. The stator was fixed onto a support of the application via six mounting holes by using M2 screws.

The rotor consisted of a stainless-steel hollow-through hub and the glass code disc with a circular scale pattern. The pattern was fabricated from chromium by the photolithography process. The glass grating disc was optically centred by the help of a digital microscope and was attached to the hub by using UV-curable adhesive. The hub was placed on the application shaft via the Ø40 H6 inner hole and fixed by using eight M2 screws. The overall height of the assembled encoder was ~8.5 mm.

#### 3.1.2. Electrical Design of the Encoder

**Figure 6:**
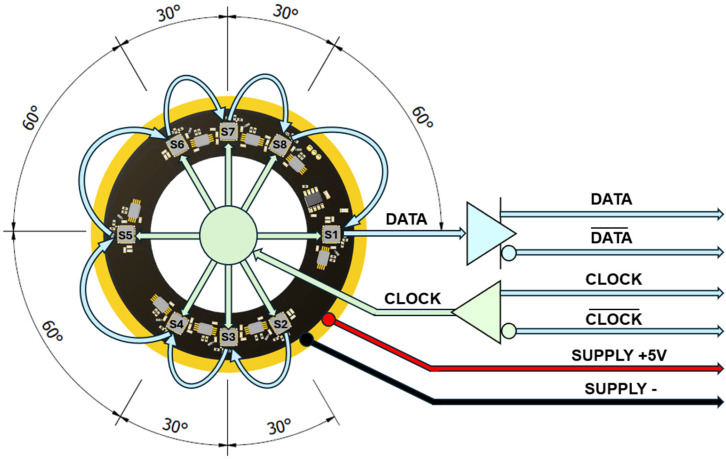
The principle electrical scheme of the developed encoder.

The measurement principle of the optical sensors was based on a reflective scanning of two circular patterns: the absolute track combined of a maximum sequence absolute data code and the incremental track with 1024 lines. A daisy-chaining of all eight optical sensors was implemented. The angular position data of sensor S1 was serially transmitted to S2, which combined the position data of both sensors and transmitted them to sensor S3, and so on. The last optical sensor S8 transmitted the combined position data from all sensors via a bidirectional serial synchronous protocol BiSS-C. Two lines of differential data from the encoder and two lines for the differential clock to the encoder were used for robust communication. To achieve higher resolution, the analogue signals produced by the optical sensors were electrically interpolated using an internal interpolator. By dividing each signal period in smaller increments, the overall system attained a final resolution of 2^22^ (4194304) unique absolute positions per one revolution. The principle electrical scheme is presented in Figure 6.

### 3.2. Cross-Calibration of the Produced Optical Encoder

**Figure 7:**
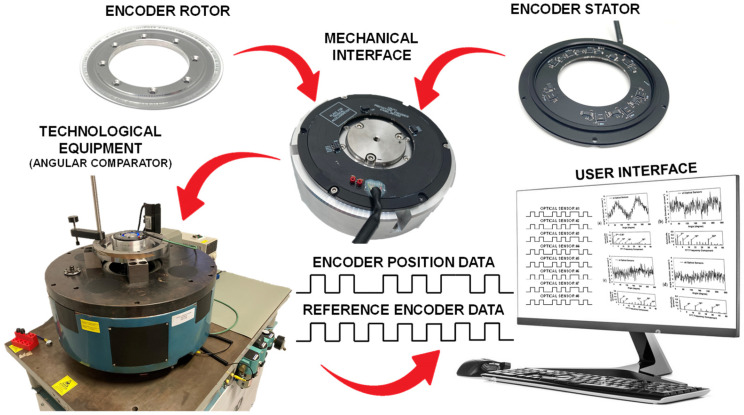
The schematic representation of the experimental research.

The cross-calibration procedure was performed to determine the actual position deviation values of each of the eight integrated optical sensors. The schematic representation of the performed experimental research is shown in Figure 7.

This procedure was performed by using calibrated technological equipment (JSC “Precizika Metrology”, Vilnius, Lithuania) that could assure a measurement uncertainty of ±0.65 arcsec. The tested encoder was mounted on the angular comparator via the special mechanical interface jig, which was designed to mimic a real installation condition. The encoder was coupled with the high-accuracy reference encoder by using air bearing. The system was rotated by a servomotor via the worm gear. The equipment was set to record the readings of tested and reference encoders at specific angular positions in a dynamic regime. The obtained data later were analysed and the differences between the positions were accepted as the measurement deviations.

**Figure 8:**
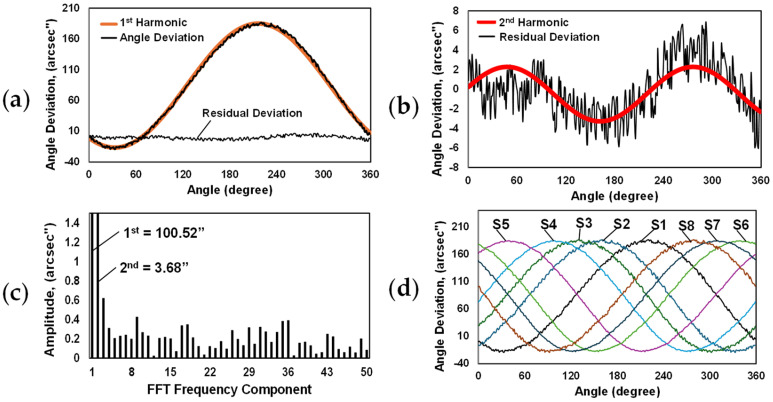
Representative plots: (**a**) deviation plot of optical sensor S1 with marked 1st harmonic and residual deviation after the elimination of the 1st harmonic; (**b**) residual deviation with marked 2nd harmonic; (**c**) Fourier harmonics of sensor S1; (**d**) angle deviations of all eight optical sensors.

The deviation plot of the optical sensor S1 is shown in Figure 8a. The span of the error reached approximately 206 arcsec. It is clearly seen that the majority of the error was caused by the 1st harmonic component, which was directly related to the eccentricity of the glass code disc. After the elimination of the 1st harmonic, the residual deviation had a significantly lower span of ~12.7 arcsec. Upon careful consideration, the biggest part of residual deviation was composed of the 2nd harmonic, as shown in Figure 8b. The 2nd harmonic of the deviation was directly related to the elliptic shape of the glass disc pattern or swash of the code disc. For a deeper analysis, fast Fourier transform was performed for the initial sensor S1 deviation data. As can be seen in Figure 8c, the first two harmonic components had the biggest amplitudes and had the greatest effect on the overall measurement accuracy of the encoder. The deviation plots of all eight sensors are presented in Figure 8d. They had the same shape but were phase-shifted with a specific angle from each other. Their nominal angular positions are marked in Figure 6. This selected arrangement of optical sensors allows us to investigate different self-calibration combinations based on the EDA method.

### 3.3. Self-Calibration of the Developed Optical Encoder

Based on a recent literature review, the equal division average (EDA) method using uniform distribution of optical sensors has been identified as the most effective approach for self-calibration in optical encoders. The EDA-method-based encoder calculates the self-calibrated absolute position $Q_{C}$ by averaging readings from multiple optical sensors evenly spaced around the full 360-degree rotation of the encoder. This position can be calculated according to the following formula:

(3) $${Q_{C}}_{i}=\frac{1}{N_{S}}\sum_{j=1}^{N_{S}}Q_{i,j},$$

where $i(i=1,2,\ldots ,N_{P})$ is the nominal absolute position, $N_{P}$ indicates the number of total unique absolute positions per one revolution. $Q_{i,j}$ is the absolute angular position transmitted by the optical sensor $S_{j}$, where $j\left(j=1,2,\ldots ,N_{S}\right)$. $N_{S}$ indicates the total number of optical sensors. This averaging procedure of position data from multiple absolute sensors effectively reduces periodic errors and harmonics. Specifically, this distribution allows the elimination of integral multiples of $N_{S}$-order Fourier components of the position deviation curve produced by each optical sensor. By spacing the sensors evenly, each position deviation is equally weighted, which smooths out systematic errors and reduces the impact of mechanical inaccuracies. To effectively compensate for grating eccentricity, a minimum of two optical sensors arranged 180 degrees apart is required. This configuration helps in reducing the amplitude of eccentricity related to the 1st harmonic by capturing data from opposite points on the disc. To correct for code disc inclination, at least three optical sensors are necessary. This arrangement allows for a more accurate measurement and the compensation of angular deviations caused by the tilt of the disc, which manifests as the 2nd harmonic.

Higher-order Fourier harmonics, as illustrated in Figure 8c, may be associated with mechanical irregularities or distortions in the optical components, such as deformations of the code disc or imperfections in the grating structure, leading to variations in the measured position. Additionally, harmonics related to high-frequency errors, such as those arising from sub-division inaccuracies, can also be significant and exhibit high amplitudes. Ignoring these harmonics can be crucial for achieving accurate and reliable measurements. Using more sensors improves the accuracy of error compensation by providing more data points for averaging. Additional sensors offer better coverage of the encoder`s full rotation, allowing for the more effective detection of various error sources. In order to reduce more Fourier components, a self-calibrated position $Q_{C}$ could be calculated by averaging the position readings of multiple sets of sensors as follows:

(4) $${Q_{C}}_{i}=\frac{1}{N_{Set}}\sum_{n=1}^{N_{Set}}\left(\frac{Q_{i,1}+\sum_{j=1}^{N_{S}}Q_{i,j}}{N_{S}}\right),$$

where: $n\left(n=1,2,\ldots ,N_{set}\right)$ is a number of the set. $N_{Set}$ corresponds to the total number of sets.

**Figure 9:**
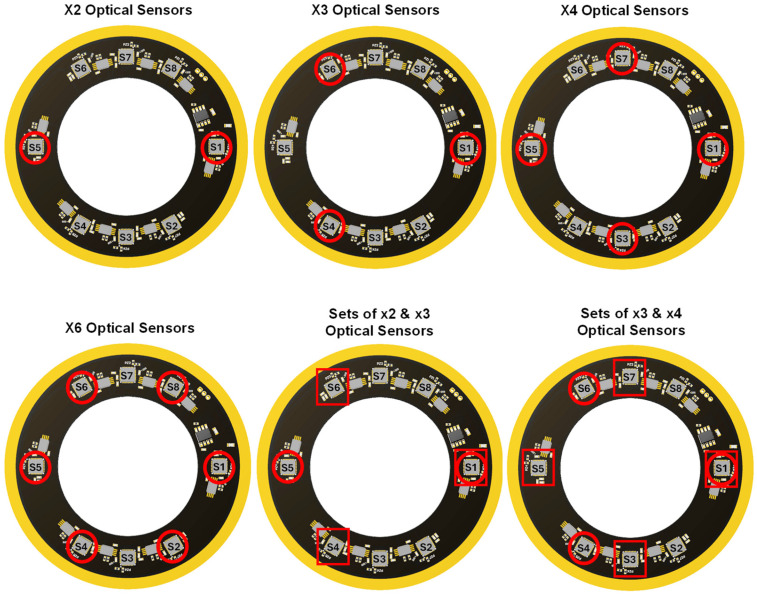
Conception diagram of the specific optical sensor used in different self-calibration arrangements. Where red circles indicate the optical sensors included in each arrangement, and red squares help to distinguish optical sensors that belong to different set.

Considering the specific positions of optical sensors on the produced encoder, and taking sensor S1 as the common one, the following combinations of self-calibration could be investigated: four arrangements of equally spaced ×2, ×3, ×4, and ×6 optical sensors, as well as two multicombinations of ×2 and ×3, and ×3 and ×4 sets. Conceptual diagrams of the analysed arrangements are shown in Figure 9.

## 4. Experimental Results

**Figure 10:**
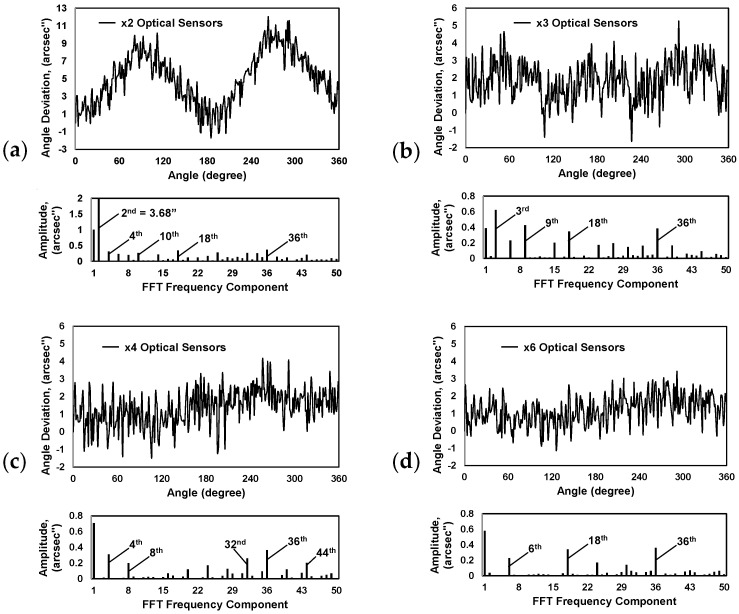

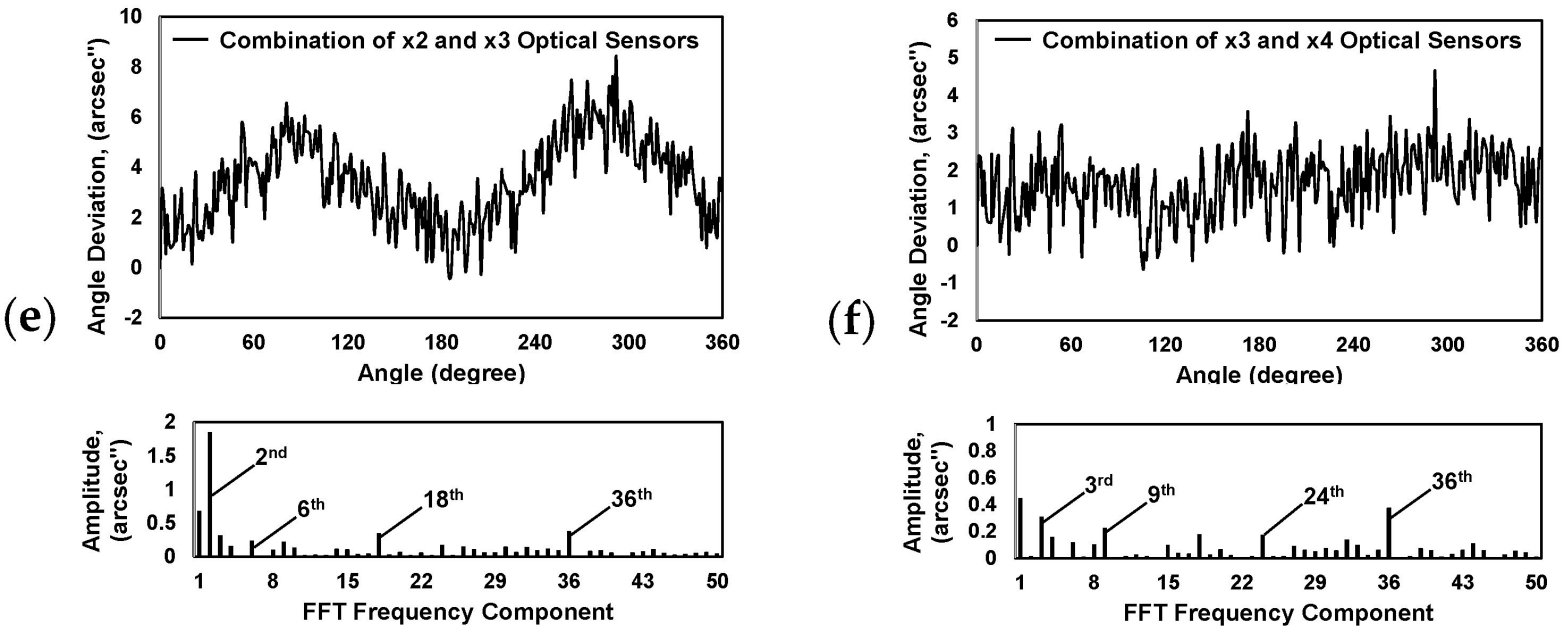
Obtained plots of angle deviation and Fourier harmonics of (**a**) ×2 optical sensors; (**b**) ×3 optical sensors; (**c**) ×4 optical sensors; (**d**) ×6 optical sensors; (**e**) multicombination of ×2 and ×3 optical sensors; (**f**) multicombination of ×3 and ×4 optical sensors.

The absolute angular position values of the self-calibrating encoder were calculated by taking the recorded readings of the corresponding optical sensors and using Equations (3) and (4). Angle deviations were obtained by comparing these calculated values with the readings of the reference encoder. Deviation plots and their Fourier harmonic compositions are presented in Figure 10.

The encoder with two optical sensors (S1 and S5) arranged at an angle of 180 degrees to each other had an angle deviation span of approximately 13.7 arcsec (see Figure 10a). This combination allowed the reduction of the amplitude of the 1st harmonic up to 0.99 arcsec and significantly improved the total accuracy. However, the specifics of using two sensors did not allow the elimination of all even harmonics. The 2nd harmonic, which reached ~3.7 arcsec, had a great influence.

The angle deviation span using three evenly spaced optical sensors (S1, S4, and S6) reached approximately 6.9 arcsec, as shown in Figure 10b. Such an arrangement significantly reduced the main 1st and 2nd harmonics, but did not eliminate the 3rd and its multiples, like the 9th, 18th, or 36th harmonics, which remained essential.

If four optical sensors were used (S1, S3, S5, and S7), the deviation span was reduced up to ~5.7 arcsec (see Figure 10c). Although the influence of the code disc eccentricity (1st harmonic) was not as effectively compensated, when using three optical sensors (its amplitude reached 0.7 arcsec), this arrangement significantly eliminated the amplitudes of the 2nd and 3rd harmonics. The 4th harmonic and its multiples, such as the 8th, 32nd, 36th, or 44th, remained decisive.

The self-calibratable encoder with six optical sensors (S1, S2, S4, S5, S6, and S8), evenly spaced every 60 degrees, had a measurement deviation span of ~4.6 arcseconds. This arrangement demonstrated the most efficient elimination of the first three Fourier components. The 6th harmonic and its multiples, the 18th and 36th, had the greatest influence on the rest of the deviation. See Figure 10d.

Two multicombinations were tested, which included two sets of equally spaced sensors: ×2 and ×3 (S1, S5 and S1, S4, S6) as well as ×3 and ×4 (S1, S4, S6 and S4, S3, S5, S7). Their peak-to-peak values of angular position measurement deviation were 8.9 arcsec and 5.3 arcsec, respectively. The first multicombination could not compensate for the 2nd harmonic well. Its remaining amplitude was 1.8 arcseconds. The 6th, 18th, and 36th harmonics were dominant, as can be seen in Figure 10d. The second multicombination, shown in Figure 10e, eliminated the first three harmonics well. However, it required six different optical sensors, and comparing to the arrangement of the equally spaced six sensors, its total range of deviation was greater.

## 5. Discussion and Conclusions

In this research, the self-calibratable absolute modular optical encoder was developed. Its open-type design had many advantages compared to closed-type rotary encoders, like compact size, low height, and no wearing parts. The advanced reflective optical sensors allowed for operation under wide tolerances, making it easy to install. However, the main issue with such a type of encoder remained its accuracy, which was sensitive to the mechanical installation of the rotor. The performed cross-calibration versus the reference encoder showed that the eccentricity of the code disc and other mounting errors, such as disc inclination, had the greatest influence on the accuracy and constituted the largest part of the measured deviation.

The equal division average (EDA) method was used to overcome this problem by integrating multiple optical sensors in the stator of the encoder. A self-calibrated absolute position could be calculated by taking readings from appropriately arranged sensors and transmitted in real time, as soon as the encoder was turned on without any angular motion. The produced prototype encoder had eight specifically positioned optical sensors in order to experimentally investigate the effectiveness of self-calibration using a different number of sensors and their combinations.

The obtained results showed that the applied method was very effective in eliminating mounting errors of the encoder. The measurement deviation was reduced by more than 90% by integrating a second optical sensor. The overall accuracy of the encoder improved as the number of sensors increased. However, any further increase in sensors or the use of their multicombinations must be carefully and rationally considered to evaluate the benefits relative to the specific application. The lowest peak-to-peak angle deviation (~4.6 arcsec) and the ability to efficiently reduce the biggest first three harmonics were demonstrated by the arrangement of six equally spaced optical sensors. Combining the sets of these optical devices into the tested multicombinations did not demonstrate a greater advantage in overall accuracy.

After summarizing the obtained results, the following general conclusions can be drawn:

1. Applying a self-calibration method by integrating additional optical sensors in a modular-type optical encoder is an effective approach to eliminate its mounting errors.
2. In this way, a high accuracy of the encoder is ensured, while maintaining easy installation and all the advantages of the modular kit encoder.

A further direction of research could be related to self-calibration efficiency studied under real environmental conditions.

## Data Availability

The original contributions presented in the research are included in the article, further inquiries can be directed to the authors.

## References

[1] Vazques-Gutierrez Y., O’Sullivan D.L., Kavanagh R.C. (2019). Study of the impact of the incremental optical encoder sensor on the dynamic performance of velocity servosystems. J. Eng..

[2] Vazques-Gutierrez Y., O’Sullivan D.L., Kavanagh R.C. (2020). Small-signal modelling of the incremental optical encoder for motor control. IEEE Trans. Ind. Electron..

[3] Zhang Z., Olgac N. (2018). Zero magnitude error tracking control for servo system with extremely low-resolution digital encoder. Int. J. Mechatron. Manuf. Syst..

[4] Algburi R.N.A., Gao H. (2019). Health assessment and fault detection system for an industrial robot using the rotary encoder signal. Energies.

[5] Li P., Liu X. (2019). Common sensors in industrial robots: A review. J. Phys. Conf. Ser..

[6] Rodriguez-Donate C., Osornio-Rios R.A., Rivera-Guillen J.R., Romero-Troncoso R.D.J. (2011). Fused smart sensor network for multi-axis forward kinematics estimation in industrial robots. Sensors.

[7] Burnashev M.N., Pavlov P.A., Filatov Y.V. (2013). Development of precision laser goniometer system. Quantum Electron..

[8] Kim J.A., Kim J.W., Kang C.S., Jin J., Eom T.B. (2013). Calibration of angle artifacts and instruments using a high precision angle generator. Int. J. Precis. Eng. Manuf..

[9] Huang Y., Xue Z., Huang M., Qiao D. (2018). The NIM continuous full circle angle standard. Meas. Sci. Technol..

[10] Pisani M., Astrua M. (2017). The new INRIM rotating encoder angle comparator (REAC). Meas. Sci. Technol..

[11] Pavlov P.A. (2017). Aspects of the cross-calibration method in laser goniometry. Meas. Tech..

[12] Pavlov P.A. (2020). A method for investigating the error of a laser dynamic goniometer. Meas. Tech..

[13] Akgoz S.A., Yandayan T. (2018). High precision calibration of polygons for emerging demands. J. Phys. Conf. Ser..

[14] Lu X.D., Trumper D.L. (2009). Self-calibration of on-axis rotary encoders. CRIP Ann..

[15] Lu X.D., Graetz R., Ammin-Shahidi D., Smeds K. (2007). On-axis self-calibration of angle encoders. CRIP Ann.-Manuf. Technol..

[16] Gou L., Peng D., Chen X., Wu L., Tang Q. (2019). A self-calibration method for angular displacement sensor working in harsh environments. IEEE Sens. J..

[17] Ellin A., Dolsak G. (2008). The design and application of rotary encoders. Sens. Rev..

[18] Yu H., Wan Q., Sun Y., Lu X., Zhao C. (2019). High precision angular measurement via dual imaging detectors. IEEE Sens. J..

[19] Ren S., Liu Q., Zhao H. Error analysis of circular gratings angle-measuring systems. Proceedings of the 2016 International Conference on Electrical, Mechanical and Industrial Engineering.

[20] Watanabe T., Fujimoto H., Nakayama K., Masuda T., Kajitani M. (2003). Automatic high-precision calibration system for angle encoder (II). SPIE Opt. + Photonics.

[21] Watanabe T., Fujimoto H., Masuda T. (2005). Self-calibratable rotary encoder. J. Phys. Conf. Ser..

[22] Watanabe T., Fujimoto H. (2009). Application of a self-calibratable rotary encoder. Proc. ISMTII.

[23] Watanabe T. (2012). Compact self-calibratable rotary encoder. J. Jpn. Soc. Precis. Eng..

[24] Watanabe T., Kon M., Nabeshima N., Taniguchi K. (2014). An angle encoder for super-high resolution and super-high accuracy using SelfA. Meas. Sci. Technol..

[25] Watanabe T., Fujimoto H., Nakayama K., Masuda T., Kajitani M. (2001). Automatic high-precision calibration system for angle encoder. SPIE Opt. + Photonics.

[26] Ueyama Y., Furutani R., Watanabe T. (2020). A super-high-accuracy angular index table. Meas. Sci. Technol..

[27] Kim J.A., Kim J.W., Kang C.S., Jin J., Eom T.B. Precision angle comparator using self-calibration of scale errors based on the equal-division-averaged method. Proceedings of the MacroScale 2011 “Recent Developments in Traceable Dimensional Measurements”.

[28] Kokuyama W., Watanabe T., Nozato H., Ota A. (2016). Angular velocity calibration system with a self-calibratable rotary encoder. Measurement.

[29] Huang Y., Xue Z., Lin H., Wang Y. (2016). Development of portable and real-time self-calibration angle encoder. Proceedings of the SPIE 9903, Seventh International Symposium on Precision Mechanical Measurements.

[30] Ishii N., Taniguchi K., Yamazaki K., Aoyama H. (2018). Super-accurate angular encoder system with multi-detecting heads using VEDA method. J. Jpn. Soc. Precis. Eng..

[31] Ishii N., Taniguchi K., Yamazaki K., Aoyama H. (2018). Development of super-accurate angular encoder system with multi-detecting heads using VEDA method. J. Adv. Mech. Des. Syst. Manuf..

[32] Masuda T., Kajitani M. (1993). High accuracy calibration system for angular encoders. J. Robot. Mechatron..

[33] Kiryanov V.P., Petukhov A.D., Kiryanov A.V. (2022). Analysis of self-calibration algorithms in optical angular encoders. Optoelectron. Instument. Proc..

[34] Ke X., Zhang M., Zhao K. (2021). Moiré fringe method via scanning transmission electron microscopy. Small Methods.

[35] S’Ari M., Koniuch N., Brydson R., Hondow N., Brown A. (2020). High-resolution imaging of organic pharmaceutical crystals by transmission electron microscopy and scanning moiré fringes. J. Microsc..

[36] Hu W.T., Tian M., Wang Y.J., Zhu Y.L. (2024). Moiré fringe imaging of heterostructures by scanning transmission electron microscopy. Micron.

[37] Prabhakara V., Jannis D., Béché A., Bender H., Verbeeck J. (2020). Strain measurement in semiconductor FinFET devices using a novel moiré demodulation technique. Semicond. Sci. Technol..

[38] Wang Q., Ri S., Xia P. (2021). Wide-view and accurate deformation measurement at microscales by phase extraction of scanning moiré pattern with a spatial phase-shifting technique. Appl. Opt..

[39] Chen R., Zhang Q., He W., Xie H.M. (2022). Orthogonal sampling moiré method and its application in microscale deformation field measurement. Opt. Lasers Eng..

[40] Zhang H., Cao Y., Li H., An H., Wu H. (2024). Spatial computer-generated Moiré profilometry. Sens. Actuators A Phys..

[41] Wang L., Cao Y., Li C., Wan Y., Li H., Xu C., Zhang H. (2021). Improved computer-generated moire profilometry with flat image calibration. Appl. Opt..

[42] Wang L., Cao Y., Li C., Wan Y., Wang Y., Li H., Xu C., Zhang H. (2021). Computer-generated moiré profilometry based on flat image demodulation. Opt. Rev..

[43] Li C., Cao Y., Wang L., Wan Y., Li H., Xu C., Zhang H. (2020). Computer-generated moiré profilometry based on fringe-superposition. Sci. Rep..

[44] Wang N., Jiang W., Zhang Y. (2020). Misalignment measurement with dual-frequency moiré fringe in nanoimprint lithography. Opt. Express.

[45] Jiang W., Wang H., Xie W., Qu Z. (2023). Lithography alignment technique based on moiré fringe. Photonics.

[46] Wang N., Jiang W., Zhang Y. (2021). Moiré-based sub-nano misalignment sensing via deep learning for lithography. Opt. Lasers Eng..

[47] Gurauskis D., Przystupa K., Kilikevičius A., Skowron M., Matijošius J., Caban J., Kilikevičienė K. (2022). Development and Experimental Research of Different Mechanical Designs of an Optical Linear Encoder’s Reading Head. Sensors.

[48] Zhu W., Lin Y., Huang Y., Xue Z. (2020). Research on sinusoidal error compensation of Moiré signal using particle swarm optimization. IEEE Access..

[49] Choi I., Yoon S.J., Park Y.L. (2024). Linear electrostatic actuators with Moiré-effect optical proprioceptive sensing and electroadhesive braking. Int. J. Robot. Res..

[50] Wu J., Zhou T.T., Yuan B., Wang L.Q. (2016). A digital Moiré fringe method for displacement sensors. Front. Inform. Technol. Electron. Eng..

[51] Walcher H. (2014). Position Sensing: Angle and Distance Measurement for Engineers.

[52] Yang Z., Ma X., Yu D., Cao B., Niu Q., Li M., Xin C. (2023). An ultracompact angular displacement sensor based on the Talbot effect of optical microgratings. Sensors.

[53] Kao C.F., Huang H.L., Lu H. (2010). Optical encoder based on the fractional Talbot effect using two-dimensional phase grating. Opt. Commun..

[54] Kao C.F., Lu H. (2005). Optical encoder based on the fractional Talbot effect. Opt. Commun..

[55] Crespo D., Alonso J., Tomas M., Eusebio B. (2000). Optical encoder based on the Lau effect. Opt. Eng..

[56] Sudol R., Thompson B.J. (1981). Lau effect: Theory and experiment. Appl. Opt..

[57] Ye G., Liu H., Xie H., Asundi A. (2016). Optimizing design of an optical encoder based on generalized grating imaging. Meas. Sci. Technol..

[58] Ye G., Liu H., Fan S., Li X., Yu H., Lei B., Shi Y., Yin L., Lu B. (2015). A theoretical investigation of generalized grating imaging and its application to optical encoders. Opt. Commun..

[59] Liu H., Ye G., Shi Y., Yin L., Chen B., Lu B. (2016). Multiple harmonics suppression for optical encoders based on generalized grating imaging. J. Mod. Opt..

[60] Ye G., Liu H., Lei B., Niu D., Xing H., Wei P., Lu B., Liu H. (2020). Optimal design of a reflective diffraction grating scale with sine-trapezoidal groove for interferential optical encoders. Opt. Lasers Eng..

[61] Liu C.H., Cheng C.H. Development of a multi-degree-of-freedom laser encoder using ±1 order and ±2 order diffraction rays. Proceedings of the 10th International Symposium of Measurement Technology and Intelligent Instruments.

[62] Wiseman K.B., Kissinger T., Tatam R.P. (2021). Three-dimensional interferometric stage encoder using a single access port. Opt. Lasers Eng..

[63] Qin S., Huang Z., Wang X. (2010). Optical angular encoder installation error measurement and calibration by ring laser gyroscope. IEEE Trans. Instrum. Meas..

[64] Li X., Ye G., Liu H., Ban Y., Shi Y., Yin L., Lu B. (2017). A novel optical rotary encoder with eccentricity self-detection ability. Rev. Sci. Instrum..

[65] Jia H.K., Yu L.D., Zhao H.N., Jiang Y.Z. (2019). A new method of angle measurement error analysis of rotary encoders. Appl. Sci..

[66] Yu H., Wan Q., Liang L., Zhao C. (2024). Analysis and elimination of grating disk inclination error in photoelectric displacement measurement. IEEE Trans. Ind. Electron..

[67] Sanchez-Brea L.M., Morlanes T. (2008). Metrological errors in optical encoders. Meas. Sci. Technol..

